# Physical exercise therapy as an anti-aging strategy for osteosarcopenia: a narrative review

**DOI:** 10.3389/fragi.2026.1791384

**Published:** 2026-05-28

**Authors:** Ting ting Liu, Pengwei Zhang, Wan tao Dong, Yan long Gong, Zhi jie Fang, Ning Li, Xin Ma

**Affiliations:** 1 Gansu University of Chinese Medicine, Lanzhou, China; 2 Affiliated Hospital of Gansu University of Chinese Medicine, Lanzhou, China

**Keywords:** cellular senescence, non-pharmacological, osteosarcopenia, physical exercise therapy, review

## Abstract

With global population aging accelerating, osteosarcopenia—the coexistence of sarcopenia and osteoporosis—has become a critical health challenge leading to frailty, falls, and disability in the elderly. This syndrome is closely linked to chronic inflammation, metabolic imbalance, and cellular aging. Physical exercise therapy, as a non-pharmacological intervention, shows unique advantages in preventing musculoskeletal degeneration and restoring metabolic homeostasis. Evidence indicates that regular aerobic and resistance exercise promotes osteogenesis and muscle protein synthesis while inhibiting bone and muscle loss through mechanical loading, regulation of myokines and osteokines, and energy metabolism remodeling. Key molecular pathways include activation of the SIRT1/AMPK/PGC-1α axis, modulation of mTOR signaling, and suppression of inflammatory cytokines such as IL-6 and TNF-α, which collectively enhance mitochondrial function and reduce oxidative stress. Moreover, physical exercise strengthens muscle–bone crosstalk via factors like irisin, myostatin, osteocalcin, and sclerostin, exerting systemic anti-aging effects. Future studies should emphasize personalized physical exercise prescriptions combined with biomarker monitoring and smart technologies to achieve sustainable musculoskeletal health and promote healthy aging.

## 1 Introduction

The acceleration of global population aging is not merely an abstract demographic trend, but a transformative process that is fundamentally reshaping public health systems and individual health management. As the proportion of the population aged 65 and older continues to rise, degenerative changes in the musculoskeletal system have evolved from natural byproducts of aging into a systemic healthcare burden. Within this context, osteosarcopenia-the co-occurrence of reduced muscle mass and function and bone density-has emerged as an important contributor to adverse outcomes This condition is primarily characterized by a progressive decline in physical strength and bone quality, which synergistically amplifies the risk of falls, delayed fracture healing, and a loss of functional independence (lynes et al., 2021; Kirk et al., 2020; Chen et al., 2024). Beyond localized tissue loss, osteosarcopenia is a complex syndrome associated with metabolic disturbances, such as reduced insulin sensitivity and chronic low-grade inflammation, which ultimately contributes to increased all-cause mortality (Huang et al., 2023; Yi et al., 2025). This suggests that interventions focusing solely on either muscle or bone may fail to address the essential “crosstalk” between these two tissues. Furthermore, traditional pharmacological treatments for osteoporosis offer limited benefits for muscle synthesis and carry risks of gastrointestinal side effects or renal strain, complicating their use in elderly patients with multiple comorbidities.

Consequently, physical exercise therapy has emerged as a safe and integrative non-pharmacological strategy. By applying physiological loading, physical exercise serves as a primary stimulus that synchronizes the adaptive remodeling of both bone and muscle (Kemmler et al., 2020). For instance, resistance training and weight-bearing aerobic exercises have been shown to improve bone density and muscle strength simultaneously. These benefits are driven by the physical exercise-induced secretion of growth-promoting signaling molecules, such as insulin-like growth factor-1 (IGF-1) and irisin (Gomez-Bruton et al., 2019). At the molecular level, physical exercise triggers key pathways—including Wnt/β-catenin and AMPK/mTOR—that act as “switches” to inhibit bone loss and promote muscle protein synthesis (Vicenti et al., 2020; Dong et al., 2025). This coordination is further supported by the activation of the protein PGC-1α, which enhances mitochondrial energy production, thereby improving muscle endurance and supporting the survival of bone cells (Ma et al., 2020). On a systemic level, physical exercise does more than improve body composition; it regulates metabolic, endocrine, and immune networks to create a multi-dimensional anti-aging effect (Westerterp, 2018; Martín-Rodríguez et al., 2024).

However, transforming physical exercise into a quantifiable clinical intervention remains challenging. Developing personalized “physical exercise prescriptions” requires balancing effectiveness with safety, particularly for frail individuals at risk of cardiovascular events or falls. Furthermore, the lack of professional guidance and equipment in some regions presents a barrier to widespread implementation. These challenges do not diminish the validity of physical exercise therapy but rather highlight the need to bridge the gap between laboratory research and clinical practice. Addressing osteosarcopenia should move beyond the search for a “magic pill” and instead prioritize physical activity as an irreplaceable physiological need, establishing a social ecology where physical exercise is central to healthy aging interventions. Therefore, this narrative review aims to clarify the pathophysiological mechanisms of osteosarcopenia and to examine how physical exercise therapy may counteract these processes. By analyzing the underlying pathways and clinical applications, we outline the mechanistic basis for physical exercise as an anti-aging intervention in this condition.

## 2 Data and methods

We searched PubMed and Web of Science for English-language articles published in the last 10 years (2016–2026) covering clinical research, basic research, and reviews on osteosarcopenia. The search strategy combined the term “osteosarcopenia” with each of the following keywords using the Boolean operator AND: treatment, epidemiology, diagnosis, pathogenesis, and review. In addition, we manually screened the reference lists of the retrieved articles for relevant studies. A total of 2,587 records were initially identified. After deduplication, two reviewers independently screened titles and abstracts, then assessed full texts against predefined inclusion criteria. Eligible studies included (1) original research, clinical trials, observational studies, or systematic reviews explicitly addressing osteosarcopenia in humans or animal models; and (2) foundational studies focusing on sarcopenia or osteoporosis that provided essential mechanistic insights directly applicable to the pathophysiology or therapeutic targeting of osteosarcopenia. Studies were excluded if they were conference abstracts, editorials. Disagreements were resolved by discussion. Following full-text review, 153 references were selected for inclusion in this narrative review. Of these, the majority directly address osteosarcopenia, while a subset of high-value references from the sarcopenia and osteoporosis literature are cited to contextualize shared mechanisms and intervention principles where osteosarcopenia-specific evidence remains limited. For certain foundational or seminal works predating the search window, the publication year restriction was waived to ensure comprehensive coverage of established concepts.

## 3 Pathophysiology of osteosarcopenia: from cellular hallmarks to systemic dysregulation

**FIGURE 1:**
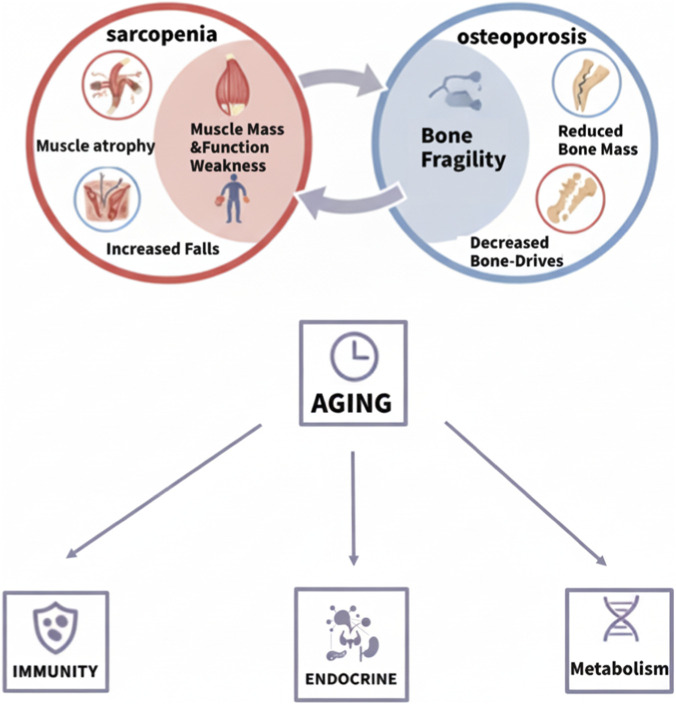
Potential mechanisms underlying Osteosarcopenia syndrome. (This schematic diagram illustrates the bidirectional relationship between sarcopenia and osteoporosis in the aging process. Aging acts as a central driving factor that disrupts systemic homeostasis and affects multiple biological systems, including immunity, endocrine regulation, and metabolism. These alterations contribute to progressive deterioration of both skeletal muscle and bone. Sarcopenia is characterized by muscle atrophy, reduced muscle mass and function, and an increased risk of falls. Osteoporosis is characterized by decreased bone mass, microarchitectural deterioration, and increased bone fragility. The two conditions interact with each other in a vicious cycle. Muscle weakness reduces mechanical loading on bone and increases fall risk, thereby accelerating bone loss and fracture susceptibility. Bone-drives represent the mechanical support and biochemical signaling provided by bone tissue that help maintain muscle function and musculoskeletal integrity, and their decline during osteoporosis contributes to the progression of sarcopenia).

Osteosarcopenia refers to the concurrent presence of osteoporosis and sarcopenia in the same individual, constituting a complex geriatric syndrome—a multifactorial condition that transcends single-organ pathology and arises from shared aging-related mechanisms, ultimately predisposing to falls, fractures, and loss of functional independence. Its core features include reduced bone mass, deteriorated bone microarchitecture, and diminished muscle mass and function. This syndrome is closely related to a significant increase in the risk of falls, fractures, chronic pain, dysfunction and death, and has become an important factor affecting the quality of life and social medical burden of the elderly (Kirk et al., 2020). Therefore, the internal and external environmental changes that affect muscle and bone metabolism during aging are often synergistic, making the degenerative changes of the two tissues present a mutually reinforcing pathological process. The potential mechanisms as shown in Figure 1.

Epidemiological studies suggest that approximately 10%–15% of individuals over 60 years of age worldwide may be affected by both osteoporosis and sarcopenia; however, these estimates vary considerably depending on the population studied, diagnostic criteria, and study design. The prevalence may increase to around 30% among adults over 80 years old, although substantial heterogeneity has been reported across studies (Hirschfeld et al., 2017; Wang et al., 2024a). The syndrome is closely related to the significant increase in the risk of falls, fractures, chronic pain, dysfunction and death, and has become an important factor affecting the quality of life and social medical burden of the elderly. At the pathological level, osteosarcopenia is not a simple superposition of the two diseases, but a systemic degenerative state driven by common molecular and cellular mechanisms. Bone and muscle are derived from mesenchymal stem cells (MSCs), both of which are regulated by similar growth factors, hormones and signaling pathways in embryonic development and adult homeostasis (Dezawa et al., 2005). Therefore, the internal and external environmental changes that affect muscle and bone metabolism during aging are often synergistic, making the degenerative changes of the two tissues present a mutually reinforcing pathological process. The aging body is often in a chronic low-grade inflammatory state, which is characterized by the continuous increase of inflammatory factors such as IL-6, TNF-α, and CRP (Tao et al., 2024). These molecules can promote osteoclast differentiation and inhibit osteoblast activity by activating NF-κB and RANKL signals in bone tissue; in skeletal muscle, it induces protein degradation and muscle atrophy (Aitella et al., 2025). At the same time, the increase of oxidative stress level leads to mitochondrial dysfunction, ROS accumulation, DNA damage and protein carbonylation, which further accelerates the apoptosis and senescence of skeletal muscle cells (Vatner et al., 2020; Papadopoulou et al., 2021).

### 3.1 Biological basis of muscle-bone coupling

Traditionally, muscles and bones have been regarded as anatomically adjacent but functionally distinct tissue systems. However, recent studies have gradually revealed that they actually form a highly integrated “muscle-skeletal coupling unit”, with their coupling not only manifesting in mechanical transmission and morphological adaptation but also extending to the bidirectional regulation of molecular signaling networks. The mechanical load generated by muscle contraction has been confirmed as the main physiological stimulus for bone formation (Rauch et al., 2004). This mechanism is not merely a simple physical pressure stress conduction but is precisely transduced through osteocytes, the key mechanical sensors: osteocytes convert external mechanical signals into intracellular biochemical instructions through integrin-mediated extracellular matrix anchoring, rapid responses of Piezo1 mechanosensitive ion channels, and nuclear-cytoplasmic shuttling of YAP/TAZ transcriptional coactivators, thereby dynamically regulating the balance between the Wnt/β-catenin osteogenic promoting pathway and the RANKL/OPG osteoclast regulatory axis (Wang et al., 2020; Uchinuma et al., 2024). In other words, the periodic tension exerted by muscle activity is not merely a passive load but a core variable actively shaping the bone microenvironment homeostasis. Regular muscle contractions can significantly enhance the proliferation and differentiation potential of osteoblasts while inhibiting the transformation of osteoclast precursor cells into mature osteoclasts, thereby maintaining a positive accumulation of net bone mass in the dynamic cycle of bone remodeling (Gomarasca et al., 2020; Kan et al., 2022). Bones themselves are not silent scaffold structures but active endocrine organs that secrete osteokines to inversely regulate the metabolic state and functional performance of muscles. For instance, osteocalcin has been proven to enhance the efficiency of glucose uptake by muscles and increase insulin sensitivity (Zhao et al., 2022). Sclerostin, although primarily known for inhibiting Wnt signaling and bone formation, may also interfere with the energy utilization efficiency of muscle fibers when expressed in muscles. RANKL, a classic osteoclast differentiation factor, has recently been found to affect mitochondrial biogenesis and oxidative phosphorylation capacity in muscle cells by acting on the RANK receptor on their surface (Nowicki and Jakubowska-Pietkiewicz, 2024; Kleber et al., 2019). As the largest glucose-consuming organ in the body, muscle contraction not only directly consumes energy but also regulates fat breakdown and hepatic glucose output by releasing IL-6, irisin, etc. Bones, on the other hand, feedback regulate insulin secretion and peripheral tissue sensitivity through factors such as osteocalcin. Together, they participate in the coordination of energy distribution throughout the body. Once this coupling is abnormal, not only does the mechanical load input sharply decrease, weakening the signal perception of osteocytes, suppressing Wnt pathway activity, and increasing the RANKL/OPG ratio, leading to a dominance of osteoclast activity, but also the spectrum of bone factors secreted by bones becomes distorted, with an increase in the proportion of carboxylated osteocalcin and a decrease in its biological activity, and an abnormal increase in sclerostin levels, further weakening the muscle’s response to insulin, forming a vicious cycle (Millar et al., 2020).

Although bones and muscles are anatomically distinct tissue systems, they exhibit a high degree of shared signaling pathways at the metabolic regulation level. Key molecules such as AMPK, mTOR, FoxO, and SIRT1 not only coordinate energy production and protein synthesis in muscle fibers but also deeply participate in the balance of osteoblast and osteoclast activities (Guan et al., 2025; Sadria and Layton, 2021); this shared mechanism originally maintains the functional coupling of the musculoskeletal system, but in the aging process, it becomes a common source of vulnerability. Mitochondrial dysfunction plays a pivotal role in this process. As the efficiency of the electron transport chain declines and reactive oxygen species accumulate, the reduction in ATP production not only weakens the contractile ability of muscle cells but also inhibits the energy-intensive processes required for bone matrix mineralization, thereby leading to a simultaneous exacerbation of muscle atrophy and bone density loss (Huang et al., 2025a). In a dynamically changing immune microenvironment, immune effector units such as macrophages and T cells that infiltrate the bone marrow cavity and intermuscular adipose tissue gradually shift towards a pro-inflammatory phenotype in the context of aging, continuously releasing inflammatory factors such as IL-1β and TNF-α, while the secretion of anti-inflammatory factors like IL-10 is relatively restricted (Calv et al., 2013). This immune imbalance activates a chronic low-grade inflammatory state, which, on the one hand, inhibits FoxO-mediated antioxidant defense through the NF-κB pathway, accelerating the positive feedback loop of mitochondrial damage; on the other hand, it directly interferes with mTOR’s fine regulation of anabolism, causing the rate of muscle protein degradation to exceed the regenerative capacity and promoting RANKL expression to drive bone resorption (Chen et al., 2025).

### 3.2 Aging hallmarks as upstream drivers of osteosarcopenia

#### 3.2.1 Cellular senescence

Cellular senescence constitutes a fundamental aging hallmark and a principal upstream driver of osteosarcopenia. It is defined as a stable state of cell-cycle arrest induced by various endogenous or exogenous stresses and is accompanied by extensive molecular and functional alterations. It was first described as a consequence of telomere shortening during repeated cell division, a process known as replicative senescence (Miwa et al., 2022). After approximately 50–70 divisions, telomeres progressively shorten and lose their protective structure, which activates tumor suppressor pathways and ultimately leads to irreversible growth arrest (Kyriacou and Lingner, 2024). Current evidence indicates that cellular senescence is not driven by a single pathway but rather by an integrated stress-response network involving DNA damage response (DDR), telomere attrition, mitochondrial dysfunction, and inflammatory signaling (Lóp et al., 2023). These mechanisms interact to form a reinforcing regulatory system that promotes the establishment and maintenance of the senescent phenotype.

A central initiating event in many senescence processes is the DNA damage response. DNA damage caused by telomere dysfunction, oxidative stress, or genomic instability activates the ATM/ATR signaling cascade, which stabilizes the tumor suppressor protein p53 and induces transcription of the cyclin-dependent kinase inhibitor p21 (Sabin et al., 2019). Activation of the p53–p21 axis leads to inhibition of cyclin-dependent kinases and transient cell-cycle arrest. In parallel, persistent stress signals activate the p16^Ink4a–Rb pathway, which suppresses CDK4/6 activity and maintains retinoblastoma protein (pRb) in a hypophosphorylated state, thereby enforcing irreversible cell-cycle arrest (Sharpless and Sherr, 2015). Experimental studies using mouse and human mesenchymal stem cells and fibroblasts have further demonstrated species-specific differences in senescence regulation. In mouse cells, senescence is largely dependent on the RB1–p16 pathway (Dimri, 2004), whereas in human cells the RB2/p130–p27–p16 signaling axis appears to play a more prominent role in regulating cell-cycle exit and senescence progression (Gallastegui et al., 2018). These findings highlight that while the p53–p21 and p16–Rb pathways constitute the core machinery of senescence, their relative contributions may vary across species and cell types. Mitochondrial dysfunction represents another key component of the senescence network. Aging mitochondria exhibit reduced membrane potential, impaired oxidative phosphorylation, and increased production of ROS. Excess ROS can directly damage DNA, proteins, and lipids, thereby amplifying the DDR pathway (Passos et al., 2010). In addition, ROS-mediated signaling can activate transcription factors such as NF-κB, promoting inflammatory gene expression and contributing to the development of the senescence-associated secretory phenotype (SASP) (Hou et al., 2017). Recent studies have further identified the cGAS–STING pathway as a critical molecular bridge linking genomic instability to chronic inflammation during senescence. In aged or stressed cells, nuclear DNA fragments generated by genomic instability, telomere dysfunction, or mitochondrial damage may accumulate in the cytoplasm. These cytosolic DNA fragments are sensed by cyclic GMP–AMP synthase (cGAS), which catalyzes the formation of cyclic GMP–AMP (cGAMP). cGAMP subsequently activates stimulator of interferon genes (STING), triggering downstream signaling pathways including TBK1–IRF3 and NF-κB (Salama et al., 2014). Activation of these pathways promotes the production of inflammatory cytokines and SASP factors. Persistent activation of the cGAS–STING–NF-κB axis therefore forms a feed-forward loop that amplifies inflammatory signaling and stabilizes the senescent phenotype. The integration of DDR signaling, mitochondrial dysfunction, and inflammatory pathways ultimately leads to systemic consequences of cellular senescence, including genomic instability, metabolic remodeling, stem cell exhaustion, and tissue degeneration. Accumulation of senescent cells has been documented in animal models and human tissues associated with aging-related musculoskeletal disorders. In skeletal muscle, senescent myocytes and satellite cells release SASP factors that impair regenerative capacity and promote muscle atrophy (Sousa-Victor et al., 2022). In bone tissue, senescent osteoblast lineage cells and bone marrow stromal cells disrupt the balance between bone formation and bone resorption, favoring osteoclast activity and bone loss (Farr et al., 2016).

Taken together, these interconnected senescence pathways create a shared pathogenic framework linking aging to osteosarcopenia. Persistent DNA damage, mitochondrial ROS production, and chronic inflammatory signaling synergistically promote the accumulation of senescent cells in both muscle and bone tissues, thereby contributing to the progressive decline in musculoskeletal integrity observed during aging.

#### 3.2.2 Mitochondrial dysfunction and oxidative stress

Mitochondrial dysfunction is a quintessential hallmark of aging that acts as an early and central driver of cellular decline in osteosarcopenia. Aging is associated with profound changes in mitochondrial function, including reduced oxidative phosphorylation efficiency, impaired mitochondrial biogenesis, and altered lipid metabolism. These alterations lead to decreased ATP production and excessive generation of ROS, creating a metabolic environment characterized by oxidative stress and impaired cellular homeostasis (An et al., 2019). Evidence from both animal models and cellular studies indicates that mitochondrial dysfunction represents one of the earliest metabolic events contributing to musculoskeletal aging.

Mitochondria play a fundamental role in maintaining energy supply in highly metabolically active tissues such as skeletal muscle and bone. In skeletal muscle, mitochondrial dysfunction reduces ATP availability and compromises muscle fiber contractile capacity. Cellular studies have shown that decreased mitochondrial activity alters the intracellular ATP/AMP ratio, which normally activates the energy sensor AMPK (Herzig and Shaw, 2018). Under physiological conditions, AMPK activation promotes metabolic adaptation by enhancing mitochondrial biogenesis and fatty acid oxidation through its downstream target peroxisome proliferator-activated receptor-γ coactivator-1α PGC-1α (Liang and Ward, 2006). However, aging is associated with a progressive decline in AMPK activity, which limits PGC-1α–mediated mitochondrial renewal and ultimately leads to impaired energy metabolism and muscle weakness (Milan et al., 2026). Similar metabolic disturbances occur in bone tissue, where insufficient ATP production in osteoblasts and osteocytes reduces bone matrix synthesis and promotes apoptosis, thereby weakening bone formation capacity. A major consequence of mitochondrial dysfunction is the excessive accumulation of ROS. Although low levels of ROS participate in physiological signal transduction, sustained ROS overproduction induces oxidative stress that damages proteins, lipids, and mitochondrial DNA. Experimental studies have demonstrated that elevated ROS levels activate stress-responsive signaling pathways such as NF-κB, p38 MAPK, and JNK (Forrester et al., 2018). These pathways simultaneously influence both bone and muscle metabolism. In bone tissue, oxidative stress suppresses osteoblast differentiation while promoting osteoclast formation, partly through inhibition of osteogenic transcription factors such as Runx2 and Osterix (Zulfiqar et al., 2026). In skeletal muscle, ROS-mediated signaling activates the ubiquitin–proteasome system and autophagy-lysosomal pathways, accelerating muscle protein degradation and contributing to muscle atrophy (Romanello and Sandri, 2016). Together, these processes illustrate how oxidative stress serves as a shared molecular mechanism linking metabolic dysfunction with musculoskeletal degeneration.

#### 3.2.3 Gut microbiota dysbiosis

The gut microbiome has emerged as a critical aging hallmark that influences musculoskeletal health through systemic metabolic and immune crosstalk. During aging, the diversity and composition of gut microbiota undergo significant alterations, often characterized by a reduction in beneficial bacteria and an increase in potentially pathogenic microorganisms (Uan et al., 2022). Accumulating evidence suggests that gut microbiota dysbiosis plays a crucial role in the pathogenesis of osteosarcopenia, a condition characterized by the concurrent decline of bone mass and skeletal muscle function. The gut microbiota has emerged as an important regulator of musculoskeletal health through the gut–muscle–bone axis, which integrates immune, metabolic, and endocrine signaling pathways.

The intestinal microbiota is composed of trillions of microorganisms that maintain host physiological homeostasis by regulating nutrient metabolism, immune responses, and intestinal barrier integrity. This dysbiotic state can impair intestinal barrier function and increase gut permeability, allowing microbial metabolites and endotoxins such as LPS to enter systemic circulation and trigger chronic low-grade inflammation. Persistent inflammatory signaling subsequently promotes osteoclast activation, inhibits osteoblast differentiation, and accelerates skeletal muscle protein degradation, thereby contributing to musculoskeletal deterioration (Picca et al., 2018). In addition, gut microbiota dysbiosis can affect musculoskeletal homeostasis through microbial metabolites and metabolic pathways. Short-chain fatty acids (SCFAs), including acetate, propionate, and butyrate, are important metabolites produced by microbial fermentation of dietary fibers (Lucas et al., 2018). These metabolites regulate immune responses, maintain intestinal barrier function, and modulate bone remodeling and muscle metabolism. Reduced production of SCFAs in dysbiotic states has been associated with increased inflammation, impaired osteoblast activity, and reduced skeletal muscle protein synthesis. Moreover, alterations in microbial metabolic pathways, including amino acid metabolism and bile acid metabolism, may further disrupt energy homeostasis and musculoskeletal function. Recent systematic analyses have also identified distinct microbial signatures associated with sarcopenia and osteoporosis, supporting the concept that both conditions share common microbiome-related mechanisms (Wang et al., 2017). For example, enrichment of genera such as *Eggerthella* and depletion of beneficial taxa including members of the *Lachnospiraceae* family and the genus *Blautia* have been observed in individuals with musculoskeletal decline (Liu et al., 2021; Azzolino et al., 2025). These microbial alterations may influence metabolic pathways such as purine, pyrimidine, and sulfur-containing amino acid metabolism, thereby contributing to the pathophysiology of osteosarcopenia.

Collectively, current evidence suggests that gut microbiota dysbiosis contributes to osteosarcopenia through multiple interconnected mechanisms, including intestinal barrier disruption, chronic inflammation, altered microbial metabolites, and impaired gut-muscle-bone signaling. Understanding the role of the gut microbiota in musculoskeletal aging may therefore provide novel insights into the pathogenesis of osteosarcopenia and offer potential therapeutic targets for prevention and intervention.

### 3.3 Systemic physiological axes amplifying musculoskeletal decline

#### 3.3.1 Immune dysregulation and inflammaging

Immune aging operates as a principal systemic amplifier of osteosarcopenia, converting localized cellular damage into a chronic, low-grade inflammatory state that pervades both bone and muscle compartments. Osteosarcopenia is increasingly recognized not only as a degenerative disorder of bone and skeletal muscle but also as a systemic condition closely associated with age-related immune dysregulation. Aging profoundly alters immune homeostasis, leading to reduced immune competence, persistent low-grade inflammation, and an imbalance in cytokine signaling. These alterations collectively create a chronic inflammatory microenvironment that disrupts the coordinated regulation of bone remodeling and muscle maintenance. This interaction is often conceptualized as the immune-bone-muscle axis, in which immune signaling acts as a key mediator linking musculoskeletal aging with systemic immune dysfunction.

A central feature of immune aging is immunosenescence, characterized by impaired adaptive immune responses and altered innate immune function. Human studies have demonstrated that aging is associated with reduced thymic output of naïve T cells, decreased T-cell receptor diversity, and diminished B-cell antibody production (Mittelbrunn and Kroemer, 2021). In parallel, NK cell cytotoxicity is often impaired. These changes contribute to a state of chronic immune activation known as inflammaging, which exposes musculoskeletal tissues to persistent low-grade inflammatory stimuli (Liu et al., 2023). Clinical observations have consistently shown that circulating inflammatory mediators—including IL-6, TNF-α, C-reactive protein, and IL-1β—are significantly elevated in older adults and correlate with reduced bone mineral density and decreased muscle mass (Cesari et al., 2004). At the molecular level, these inflammatory cytokines influence both muscle and bone metabolism through shared signaling pathways. In skeletal muscle, pro-inflammatory cytokines activate catabolic signaling cascades such as NF-κB and JAK/STAT3, which stimulate proteolysis and promote muscle fiber atrophy (Yin et al., 2024). In bone tissue, the same inflammatory signals enhance RANKL expression and osteoclast differentiation while suppressing osteoblast function, thereby shifting bone remodeling toward net bone resorption. Together, these processes provide a mechanistic explanation for the parallel degeneration of muscle and bone observed in osteosarcopenia (Jiang et al., 2021).

T cells play a particularly important role in this immune–musculoskeletal interaction. During aging, thymic involution leads to reduced production of naïve T cells and expansion of senescent memory T-cell populations, especially CD8^+^CD28^−^ T cells (Nikolich-Žugich, 2018). Both human and animal studies indicate that these senescent T cells exhibit a pro-inflammatory phenotype and produce large amounts of cytokines such as IFN-γ, TNF-α, and IL-17 (Pereira and Akbar, 2016). These cytokines directly stimulate osteoclastogenesis and contribute to muscle catabolism, thereby promoting musculoskeletal degeneration. Recent experimental studies have further highlighted the role of inflammasome activation as a molecular bridge connecting immunosenescence, chronic inflammation, and tissue degeneration. Inflammasomes are intracellular multiprotein complexes that detect cellular stress signals and initiate inflammatory responses. Among them, the NLRP3 inflammasome has been extensively studied in both animal models and cellular experiments of osteoporosis and sarcopenia. Activation of NLRP3 leads to caspase-1 activation and the maturation of pro-inflammatory cytokines such as IL-1β and IL-18 (Guo et al., 2015). In bone tissue, this process enhances osteoclast differentiation and suppresses osteoblast activity, accelerating bone loss. In skeletal muscle, NLRP3 activation contributes to mitochondrial dysfunction, myofiber damage, and impaired muscle regeneration (Slavin et al., 2024).

Innate immune cells also play a critical role in regulating the bone–muscle microenvironment. Macrophages, for example, exhibit remarkable phenotypic plasticity and can differentiate into pro-inflammatory M1 or anti-inflammatory M2 phenotypes depending on environmental signals. Experimental studies indicate that aging and metabolic stress favor M1 polarization. M1 macrophages produce inflammatory cytokines such as TNF-α, IL-1β, and IL-6, which activate NF-κB and JNK signaling pathways in skeletal muscle, suppress satellite cell proliferation, and impair muscle regeneration (Park et al., 2017). In bone tissue, M1 macrophages promote osteoclastogenesis through increased RANKL signaling while inhibiting osteogenic pathways associated with M2 macrophages, including BMP-2 and VEGF signaling (Tanabe et al., 2021). These immune mechanisms operate within a broader network of bidirectional communication between bone and muscle. Bone-derived factors such as osteocalcin, RANKL, and sclerostin have been shown—primarily in animal and cellular studies—to influence muscle metabolism and regeneration. Osteocalcin enhances glucose uptake and mitochondrial function in myocytes, whereas RANKL and sclerostin may impair muscle regeneration and promote inflammatory signaling. The immune microenvironment strongly modulates these cross-tissue signals, determining whether musculoskeletal tissues undergo adaptive repair or progressive degeneration (Gao et al., 2025).

Collectively, current evidence indicates that immunosenescence, chronic inflammation, inflammasome activation, and immune cell polarization form an integrated pathogenic network that drives osteosarcopenia. Through sustained inflammatory signaling and dysregulation of the immune–bone–muscle axis, aging-related immune dysfunction contributes to the coordinated decline of skeletal muscle and bone integrity.

#### 3.3.2 Metabolic and energy-sensing disruption

Beyond the intrinsic mitochondrial defects described above, aging disrupts the systemic metabolic and energy-sensing networks that coordinate tissue-level homeostasis. Metabolic dysregulation is increasingly recognized as a central driver of osteosarcopenia, linking aging-related alterations in cellular energy metabolism with the progressive degeneration of bone and skeletal muscle (Palma et al., 2024).

At the systems level, the AMPK-SIRT1-PGC-1α signaling axis represents a central regulatory network coordinating cellular energy metabolism, mitochondrial homeostasis, and antioxidant defenses. AMPK functions as a key energy sensor that is activated under conditions of low cellular energy. SIRT1, a NAD^+^-dependent deacetylase, interacts with AMPK signaling to regulate metabolic adaptation and stress resistance by modulating PGC-1α activity (Tang, 2016). Animal and cellular studies have shown that aging is associated with a progressive decline in this signaling axis. Reduced AMPK activation weakens cellular responses to metabolic stress, decreased SIRT1 activity diminishes antioxidant defenses, and impaired PGC-1α signaling limits mitochondrial biogenesis (Yoshino et al., 2018). These alterations collectively result in reduced ATP production, increased ROS accumulation, and enhanced cellular senescence in musculoskeletal tissues. In bone specifically, experimental studies have demonstrated that impaired AMPK/SIRT1 signaling inhibits osteoblast differentiation while promoting adipogenic differentiation of bone marrow mesenchymal stem cells, thereby contributing to bone loss (Hirschfeld et al., 2017). Restoration of this pathway has been shown to improve bone density and mechanical strength in animal models.

From a systems-biology perspective, osteosarcopenia therefore emerges as a multi-organ degenerative network in which metabolic dysregulation interacts with immune and endocrine alterations. Aging-associated hormonal decline, chronic inflammation, and impaired cellular energy metabolism collectively disrupt the functional coupling between bone remodeling and muscle regeneration. Reduced muscle mass decreases mechanical loading on bone, accelerating skeletal loss, while bone deterioration further limits physical activity and exacerbates muscle wasting. Molecular studies suggest that these processes are accompanied by suppression of anabolic signaling pathways such as Wnt/β-catenin and increased activation of stress-responsive pathways including FoxO transcription factors, alongside reduced expression of antioxidant genes such as SOD2 and GPx (Guan et al., 2015; Lin et al., 2016).

Taken together, current evidence indicates that mitochondrial dysfunction, oxidative stress, and disruption of the AMPK-SIRT1-PGC-1α metabolic network form a central pathogenic axis in osteosarcopenia. Through their combined effects on energy metabolism, inflammatory signaling, and cellular senescence, these metabolic alterations drive the coordinated deterioration of bone and skeletal muscle during aging.

#### 3.3.3 Hormonal changes and endocrine imbalance

Hormonal deficiency exemplifies how systemic endocrine disruption can precipitate and accelerate the musculoskeletal manifestations of aging. Hormonal plays a fundamental role in maintaining musculoskeletal homeostasis by coordinating endocrine, immune, and metabolic signaling pathways. In postmenopausal women, the rapid decline in circulating estrogen levels represents a major pathogenic driver of osteosarcopenia. Epidemiological studies in humans consistently demonstrate that menopause is associated with accelerated bone loss, reduced muscle mass, and increased incidence of frailty-related musculoskeletal disorders. These clinical observations suggest that estrogen deficiency acts as a systemic trigger that disrupts the functional coupling between bone remodeling and muscle maintenance.

At the molecular level, estrogen regulates inflammatory signaling through estrogen receptor (ER)-mediated pathways. Under physiological conditions, estrogen suppresses the activation of pro-inflammatory transcription factors such as NF-κB and limits the production of inflammatory cytokines including TNF-α, IL-1β, and IL-6. Experimental studies in animal models have shown that estrogen depletion leads to activation of inflammatory signaling cascades and increased circulating cytokine levels, thereby creating a chronic low-grade inflammatory environment (Manolagas et al., 2013). This inflammatory milieu exerts coordinated effects on both bone and skeletal muscle. In bone tissue, inflammatory cytokines promote osteoclast differentiation while inhibiting osteoblast activity, shifting the balance of bone remodeling toward net bone resorption. In skeletal muscle, persistent inflammatory signaling accelerates proteolytic pathways and contributes to muscle protein degradation. Together, these processes provide a mechanistic explanation for the simultaneous deterioration of bone and muscle observed after menopause (Manolagas and Jilka, 1995). In addition to its immunomodulatory functions, estrogen also plays an important role in protecting cellular homeostasis and mitochondrial function. Cellular and animal studies have demonstrated that estrogen deficiency increases oxidative stress by impairing mitochondrial metabolism and promoting excessive production of ROS. Elevated ROS levels trigger DNA damage responses and activate classical cellular senescence pathways, including the p53/p21 and p16^Ink4a^/Rb signaling axes (Ribas et al., 2011). Activation of these pathways results in irreversible cell-cycle arrest and accumulation of senescent cells in musculoskeletal tissues. Senescent cells subsequently secrete a range of pro-inflammatory cytokines, chemokines, and matrix-degrading enzymes collectively known as the SASP. These SASP factors amplify inflammatory signaling and further disrupt the regenerative capacity of bone and muscle cells (Wasner et al., 2022).

Recent mechanistic studies have further suggested that estrogen deficiency may activate innate immune sensing pathways that link genomic instability with chronic inflammation. Cellular stress induced by estrogen loss can lead to mitochondrial damage and leakage of DNA fragments into the cytoplasm (Decout et al., 2021). These cytoplasmic DNA fragments are recognized by the cGAS–STING signaling pathway. Activation of cGAS–STING subsequently triggers downstream TBK1–IRF3 and NF-κB signaling, promoting the production of inflammatory cytokines and SASP factors (Wasner et al., 2022). Evidence from experimental models indicates that this pathway acts as a critical mediator connecting DNA damage responses with chronic inflammatory activation during aging. Importantly, these mechanisms do not function independently but form an interconnected regulatory network linking estrogen deficiency with immunosenescence, oxidative stress, and cellular senescence. Loss of estrogen signaling increases ROS production and mitochondrial dysfunction, which activates inflammatory and DNA damage pathways (Zhao et al., 2025). These processes promote the accumulation of senescent cells and SASP secretion, thereby reinforcing chronic inflammation. The resulting inflammatory and oxidative environment further accelerates musculoskeletal degeneration by impairing osteogenic and myogenic regenerative capacity.

In addition, parathyroid hormone (PTH) is an important hormone secreted by the parathyroid glands, which plays a key role in regulating calcium and phosphorus metabolism and maintaining bone metabolism balance in the body. The main action pathways of PTH include bone tissue, kidneys, and intestines, thereby maintaining the calcium and mineral homeostasis of the body. Studies have shown that PTH can stimulate osteoblasts to express RANKL by binding to PTH1R on osteoblasts and activating the cAMP/PKA pathway in conjunction with the PKC/MAPK/NF-κB pathway, thereby promoting osteoclast differentiation and enhancing the bone resorption process. Therefore, patients with primary hyperparathyroidism have significantly reduced bone density and an increased risk of fractures (Ertürk et al., 2026). However, under intermittent low-dose administration, PTH can promote bone formation. Studies have shown that intermittent PTH stimulation of the PTH1R-cAMP/PKA pathway, as well as the activation of Wnt/β-catenin and Runx2, promotes osteoblast differentiation and bone matrix synthesis. Additionally, it inhibits osteoblast apoptosis through the PI3K/Akt and ERK pathways (Silva and Bilezikian, 2015). Moreover, PTH can indirectly affect bone metabolism by regulating calcium and phosphorus reabsorption in the kidneys and promoting calcium absorption in the intestines. It enhances intestinal calcium absorption by promoting the production of active vitamin D in the kidneys (Chen et al., 2021). Notably, PTH also has a regulatory effect on skeletal muscle. Skeletal muscle cells express PTH1R, and continuous high levels of PTH can induce muscle protein breakdown, inhibit myocyte differentiation, and be associated with the occurrence of sarcopenia; while intermittent low-dose PTH may improve muscle mass through the IGF-1/Akt pathway (Nguyen et al., 2025). Thus, it can be seen that there is a close synergistic relationship between PTH and vitamin D, both of which are involved in the regulation of mineral metabolism in the body. Vitamin D is an important substance for maintaining bone mineralization and bone metabolism. By binding to the vitamin D receptor, it regulates the expression of multiple genes, thereby participating in the regulation of calcium and phosphorus metabolism and bone tissue metabolism. Vitamin D is the main promoting pathway for calcium and phosphorus absorption in the intestines, providing a necessary foundation for bone mineralization. When vitamin D levels are insufficient, intestinal calcium absorption capacity decreases, blood calcium levels drop, and the body increases PTH secretion, thereby exacerbating bone resorption (Pike et al., 2023). On the other hand, vitamin D itself has a dual regulatory effect on bone remodeling. Vitamin D forms a heterodimer with the vitamin D receptor and retinoid X receptor in osteoblasts, recognizes the vitamin D response element (VDRE) in the promoter region, enhances the transcriptional activity of Runx2, and promotes transcription (Pike and Christakos, 2017). Conversely, vitamin D can also induce RANKL transcription through the functional VDRE in the RANKL gene promoter region, promoting osteoclast differentiation (Skalny et al., 2024). Additionally, vitamin D plays a key role in immune regulation. It can inhibit T cell activation and reduce the release of inflammatory factors, thereby reducing osteoclast generation (Liu et al., 2025a). In the muscle system, vitamin D directly acts on the nuclear receptors of muscle cells, regulating the expression of myogenic regulatory factors MyoD and myogenin, promoting muscle cell proliferation and differentiation; vitamin D deficiency can lead to muscle fiber atrophy, decreased muscle strength, and an increased risk of sarcopenia (Agoncillo et al., 2023). Clinical studies have shown that the elderly population is limited by skin synthesis capacity and nutritional deficiencies, resulting in significant vitamin D deficiency, which is highly correlated with decreased bone density, reduced muscle mass, and increased fracture risk (Giustina et al., 2023). Therefore, vitamin D supplementation has become one of the important measures for the prevention and treatment of osteoporosis and sarcopenia. Besides estrogen, PTH and vitamin D, many metabolism-related hormones are also involved in the regulation of bone metabolism, including insulin, growth hormone, IGF-1, etc. Insulin participates in the bone formation process by promoting osteoblast differentiation and enhancing bone matrix synthesis, and activates the PI3K/Akt signaling pathway to promote osteoblast proliferation and differentiation. Growth hormone and IGF-1 play a core role in bone growth and bone formation. Growth hormone can produce IGF-1 through the liver and local tissues, activate the Ras/MAPK pathway, and cooperate with the PI3K/Akt pathway to stabilize Cyclin D1, inhibit cell cycle inhibitory proteins, promote osteoblast differentiation and delay the survival cycle (Zhang et al., 2012; DiGirolamo et al., 2007). These hormones are also key regulatory factors for maintaining muscle mass. Insulin promotes muscle protein synthesis and inhibits protein degradation mediated by the ubiquitin-proteasome pathway through the PI3K/Akt/mTOR pathway; growth hormone and IGF-1 activate the Akt/mTOR and ERK pathways, promoting satellite cell activation and muscle fiber hypertrophy. Their levels decline with age and are significantly associated with sarcopenia (Mazza et al., 2024). In addition, some fat-derived hormones such as leptin and adiponectin are also involved in the regulation of bone metabolism, and they affect muscle homeostasis by regulating systemic inflammatory responses and energy metabolism: leptin resistance can promote muscle fat infiltration, while adiponectin has anti-inflammatory and muscle-protective effects (Gu et al., 2023). These metabolism-related hormones can indirectly affect bone remodeling and muscle metabolism by influencing energy metabolism and inflammatory responses, indicating the close connection between bone metabolism and the body’s metabolism and muscle system. In summary, the endocrine system participates in the dual regulation of bone metabolism and muscle metabolism through multiple hormones and signaling pathways, playing an important role in maintaining bone homeostasis and muscle mass. When these endocrine regulatory mechanisms are abnormal, the balance of bone remodeling is disrupted, and at the same time, the synthesis and decomposition of muscle are imbalanced, thereby promoting the progression of osteosarcopenia.

Taken together, current evidence from human clinical studies, animal models, and cellular experiments supports the concept that hormone deficiency acts as a central upstream regulator of osteosarcopenia. Through coordinated effects on inflammatory signaling, mitochondrial metabolism, and cellular senescence pathways, hormone disrupts the integrated bone–muscle regulatory network and ultimately drives the progressive degeneration of musculoskeletal tissues. We have summarized the pathophysiological characteristics of osteosarcopenia, as shown in Table 1.

**TABLE 1:** Pathophysiological characteristics of osteosarcopenia.

Category	Hallmark/Axis	Core mechanisms	Effects on bone	Effects on skeletal muscle	References
Upstream Drivers	Cellular Senescence	1. DNA damage response 2. p16^Ink4a–Rb pathway 3. Mitochondrial dysfunction & ROS 4. cGAS-STING-NF-κB axis 5. SASP	1. Senescent osteoblasts and bone marrow stromal cells 2. Disrupted bone formation-resorption balance (favoring osteoclast activity 3. Bone loss	1. Senescent myocytes and satellite cells 2. Impaired regenerative capacity 3. SASP-mediated muscle atrophy	Miwa et al. (2022), Kyriacou and Lingner (2024), Lóp et al. (2023), Sabin et al. (2019), Sharpless and Sherr (2015), Gallastegui et al. (2018), Passos et al. (2010), Hou et al. (2017), Salama et al. (2014), Sousa-Victor et al. (2022), Farr et al. (2016)
​	Mitochondrial Dysfunction & Oxidative Stress	1. Reduced OXPHOS & ATP production 2. Impaired mitochondrial biogenesis 3. Excessive ROS production 4. AMPK/PGC-1α signaling 5. Activation of NF-κB, p38 MAPK, JNK	1. Insufficient ATP reduces bone matrix synthesis 2. Osteoblast apoptosis 3. ROS suppresses Runx2/Osterix 4. Promotes osteoclast formation 5. Chronic low-grade inflammation promotes osteoclast activation 6. Inhibits osteoblast differentiation	1. Reduced ATP availability compromises contractile capacity 2. AMPK activity limits mitochondrial renewal 3. ROS activates ubiquitin-proteasome and autophagy-lysosomal pathways	An et al. (2019), Herzig and Shaw (2018), Liang and Ward (2006), Milan et al. (2026), Forrester et al. (2018), Zulfiqar et al. (2026), Romanello and Sandri (2016)
​	Gut Microbiota Dysbiosis	1. Reduced beneficial bacteria 2. Increased pathogens 3. Intestinal barrier disruption & LPS leakage 4. Reduced SCFAs	1. Chronic low-grade inflammation promotes osteoclast activation 2. Inhibits osteoblast differentiation	1. Inflammatory signaling accelerates muscle protein degradation 2. Reduced SCFAs impair muscle protein synthesis	Uan et al. (2022), Picca et al. (2018), Lucas et al. (2018), Wang et al. (2017), Liu et al. (2021), Azzolino et al. (2025)
Systemic Amplifiers	Immune Dysregulation & Inflammaging	1. Immunosenescence 2. Inflammaging 3. inflammasome activation 4. Macrophage M1 polarization	1. Pro-inflammatory cytokines enhance RANKL expression and osteoclast differentiation 2. Suppress osteoblast function	1. NF-κB & JAK/STAT3 activation stimulate proteolysis and myofiber atrophy 2. M1 macrophages suppress satellite cell proliferation and impair regeneration	Mittelbrunn and Kroemer (2021), Liu et al. (2023), Cesari et al. (2004), Yin et al. (2024), Jiang et al. (2021), Nikolich-Žugich (2018), Pereira and Akbar (2016), Guo et al. (2015), Slavin et al. (2024), Park et al. (2017), Tanabe et al. (2021), Gao et al. (2025)
​	Metabolic & Energy-Sensing Disruption	1. AMPK-SIRT1-PGC-1α signaling axis 2. Reduced NAD^+^ levels 3. Impaired antioxidant defenses 4. Suppressed Wnt/β-catenin; activated FoxO	1. Impaired osteoblast differentiation 2. Promotes adipogenic differentiation of bone marrow MSCs 3. Contributes to bone loss	1. Reduced mitochondrial biogenesis & ATP production 2. Increased oxidative stress and cellular senescence 3. Muscle wasting	Hirschfeld et al. (2017), Palma et al., 2024; Tang (2016), Yoshino et al. (2018), Guan et al. (2015), Lin et al. (2016)
​	Hormonal Changes & Endocrine Imbalance	1. Estrogen deficiency 2. PTH dysregulation 3. Vitamin D deficiency 4. Insulin, GH, IGF-1 5. Leptin resistance/adiponectin changes	1. Estrogen loss → NF-κB activation →osteoclastogenesis 2. Continuous high PTH 3. Vitamin D deficiency 4. bone resorption	1. oxidative stress and cellular senescence 2. muscle protein breakdown 3. MyoD, myogenin, muscle fiber atrophy 4. protein synthesis via Akt/mTOR	Manolagas et al. (2013), Manolagas and Jilka (1995), Ribas et al. (2011), Wasner et al. (2022), Decout et al. (2021), Zhao et al. (2025), Ertürk et al. (2026), Silva and Bilezikian (2015), Chen et al. (2021), Nguyen et al. (2025), Pike et al. (2023), Pike and Christakos (2017), Skalny et al. (2024), Liu et al. (2025a), Agoncillo et al. (2023), Giustina et al. (2023), Zhang et al. (2012), DiGirolamo et al. (2007), Mazza et al. (2024), Gu et al. (2023)

The abbreviations in the table mean cyclin-dependent kinase inhibitor 2A–retinoblastoma protein (p16^Ink4a–Rb). Reactive Oxygen Species (ROS), Cyclic GMP-AMP Synthase- Stimulator of Interferon Genes- Nuclear Factor Kappa-Light-Chain-Enhancer of Activated B Cells (cGAS-STING-NF-κB). Senescence-Associated Secretory Phenotype (SASP). Oxidative Phosphorylation (OXPHOS). Adenosine Triphosphate (ATP). Runt-related transcription factor 2 (Runx2). Lipopolysaccharide (LPS). Short-Chain Fatty Acids (SCFAs). Receptor Activator of Nuclear Factor-κB Ligand (RANKL). Nicotinamide Adenine Dinucleotide (NAD^+^). Forkhead box O (FoxO). Mesenchymal Stem Cells (MSCs). Parathyroid Hormone (PTH). Myogenic Differentiation (MyoD). Growth Hormone (GH).

## 4 The anti-aging mechanism of physical exercise therapy

**FIGURE 2:**
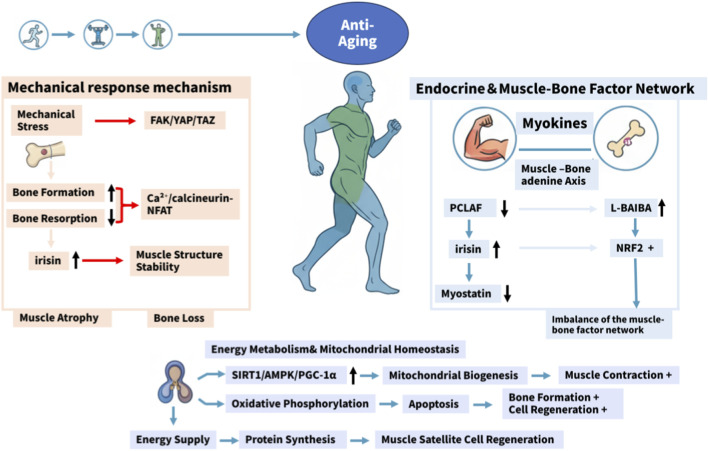
The mechanism of physical exercise therapy. The abbreviations in the figure mean calcium ion/calcineurin-nuclear factor of activated T-cells (Ca^2+^/calcineurin–NFAT). PCNA clamp associated factor (PCLAF). L-β-aminoisobutyric acid (L-BAIBA). This schematic illustrates the integrated regulatory mechanisms underlying musculoskeletal homeostasis during aging. Mechanical stimuli activate signaling pathways such as FAK/YAP/TAZ and Ca^2+^/calcineurin–NFAT to coordinate bone formation and muscle structural stability. Concurrently, the endocrine and muscle–bone factor network, including myokines and antioxidant signaling (NRF2), mediates cross-talk between skeletal muscle and bone. In addition, energy metabolism and mitochondrial homeostasis, regulated by the SIRT1/AMPK/PGC-1α axis, support mitochondrial biogenesis, protein synthesis, and satellite cell regeneration. Together, these interconnected pathways contribute to maintaining muscle–bone integrity and counteracting age-related musculoskeletal degeneration.

As a non-pharmaceutical means, physical exercise has shown a multifaceted positive effect in the prevention and treatment of osteosarcopenia, and has been recommended as the first choice for prevention and treatment. As mechanically sensitive tissues, bones and muscles rely heavily on converting external mechanical stimuli into intracellular chemical signals to regulate the balance of synthesis and catabolism. Studies have shown that the accumulation of senescent cells and the associated SASP can lead to degenerative changes in muscle and bone by triggering chronic inflammation, oxidative stress and tissue homeostasis imbalance (Fang et al., 2023; Wang et al., 2024b). This result will lead to a decrease in the sensitivity of bone to mechanical load, which requires greater stimulation to induce osteogenic response, while muscle may have abnormal mechanical signal amplification, resulting in calcium homeostasis disorder and enhanced protein degradation, exacerbating bone and muscle loss. Both sarcopenia and osteoporosis are the main factors of aging-related disability and weakness. In addition to old age, lack of physical exercise and malnutrition are also important causes of osteosarcopenia (Zhang et al., 2025). Despite heterogeneity, studies have shown that bone targeting programs have a positive effect on BMD and BMC of loaded bones, so physical exercise affects bone strength and quality in all age groups. In addition, physical exercise can also improve muscle mass, strength and function. Studies have shown that individuals with less physical exercise have a higher risk of sarcopenia (Vin et al., 2021). Therefore, regular physical exercise intervention can promote bone mass increase and bone shape optimization in childhood and adolescence, fight against muscle dysfunction and neuromuscular damage caused by aging, and have a positive impact on personal health. The mechanism of physical exercise therapy as shown in Figure 2.

### 4.1 Systematic intervention of physical exercise on osteosarcopenia

As a non-drug intervention, physical exercise therapy is considered to be one of the most feasible, cost-effective and systemic anti-aging potential strategies (Niemeijer et al., 2020). Different from drug therapy, which focuses on a single molecular target, physical exercise exerts a multi-level physiological adaptation effect on bone, muscle, immune and metabolic systems through a network of mechanical stress, metabolic regulation and secretory factors (Mamarabadi et al., 2025). For osteosarcopenia, physical exercise can not only directly enheance bone mineral density and muscle strength, but also reverse aging-related pathological processes by regulating cell energy metabolism, anti-inflammatory response and signaling pathway activity (Howe et al., 2011). In recent years, basic and clinical studies have shown that physical exercise intervention can significantly improve bone mineral content, trabecular bone structure, muscle cross-sectional area and mitochondrial function, and reduce the risk of fracture and fall (Mohebbi et al., 2023).

### 4.2 Mechanical response mechanism

Skeletal muscle and bone are highly mechanosensitive tissues whose structure and function are continuously regulated by mechanical stimuli. Regular physical activity generates mechanical forces that are sensed by mechanoreceptors in musculoskeletal cells, thereby initiating intracellular signaling cascades that coordinate tissue remodeling and metabolic adaptation. Within the context of osteosarcopenia, physical exercise can be conceptualized as a systemic intervention that restores the functional coupling between bone and muscle through integrated mechanical, metabolic, and immunological mechanisms.

From a biomechanical perspective, both clinical and experimental evidence support the principles of Wolff’s law and Frost’s mechanostat theory, which describe how bone adapts to mechanical loading (Hammer, 2015). Human studies have demonstrated that resistance training, impact loading, and vibration exercise increase bone mineral density and reduce fracture risk, particularly in postmenopausal women (Watson et al., 2019). These mechanical stimuli promote osteogenesis while suppressing bone resorption by enhancing osteoblast activity and inhibiting osteoclast differentiation. At the same time, aerobic and resistance exercise improve skeletal muscle mass and function by stimulating protein synthesis and enhancing mitochondrial efficiency (Memme et al., 2021).

At the cellular level, mechanical loading is transduced into biochemical signals through multiple mechanosensitive pathways. In skeletal muscle, mechanical stretch activates FAK and downstream YAP/TAZ signaling, promoting myofiber hypertrophy and protein synthesis (Kwon, 2024). In parallel, Ca^2+^-dependent signaling pathways such as the calcineurin–NFAT axis regulate muscle gene transcription and maintain structural integrity (Gehlert et al., 2015). In bone tissue, mechanical stimulation activates osteocytes and osteoblasts, triggering anabolic signaling pathways that favor bone formation. In contrast, the absence of mechanical stimulation leads to downregulation of these pathways, resulting in muscle atrophy and bone loss, highlighting the essential role of mechanical signals in maintaining musculoskeletal homeostasis. Importantly, the beneficial effects of physical exercise extend beyond local mechanotransduction and involve systemic regulation of inflammation and cellular senescence. Human clinical studies have shown that regular aerobic exercise significantly reduces circulating levels of pro-inflammatory cytokines, including IL-6 and TNF-α, while improving bone mineral density in middle-aged and postmenopausal women (Weng et al., 2025). Similarly, longitudinal intervention studies have demonstrated that sustained exercise programs decrease plasma levels of TNF receptors sTNFR1 and sTNFR2 and improve musculoskeletal outcomes (de Sá Souza et al., 2022). These findings indicate that physical exercise can attenuate inflammaging, a key driver of osteosarcopenia.

Evidence from animal models further supports the anti-aging effects of physical exercise at the molecular level. Moderate aerobic training in aged rodents has been shown to enhance antioxidant capacity, reduce ROS accumulation, and suppress the secretion of SASP factors (Angulo et al., 2020). Cellular studies suggest that physical exercise -induced mechanical and metabolic signals can modulate pathways involved in cellular senescence, thereby limiting the accumulation of senescent cells in bone and muscle tissues (Wang et al., 2022a). Through these mechanisms, physical exercise reduces inflammation-mediated inhibition of osteoblast differentiation and improves the regenerative capacity of skeletal muscle.

Current evidence from human clinical studies, animal experiments, and cellular research supports the concept that physical exercise functions as a form of “mechanomedicine.” Through the integrated processes of mechanical sensing, signal transduction, and gene regulation, physical exercise restores musculoskeletal homeostasis and counteracts the progression of osteosarcopenia by simultaneously targeting mechanical, metabolic, and inflammatory pathways.

### 4.3 Bidirectional regulation of endocrine and muscle-bone factor network

Skeletal muscle and bone not only produce mechanical interaction during physical exercise, but also form a complex endocrine network. Physical-exercise -induced muscle contraction causes the body to release a variety of muscle factors and bone factors. These molecules achieve cross-tissue signal communication through blood circulation and construct a “muscle-bone endocrine axis”.

During muscle contraction, various myokines such as irisin and myostatin enter the bloodstream and act on distal skeletal tissues in a hormone-like manner. Among them, irisin, a myokine produced by physical-exercise-induced cleavage of FNDC5, promotes osteoblast differentiation and bone formation, while inhibiting osteoclast formation and improving mitochondrial function via activation of the AMPK-PGC-1α pathway (Colaianni et al., 2015). Under physical exercise regulation, myostatin expression is significantly downregulated, thereby relieving its inhibitory effects on muscle protein synthesis and bone formation. This effect occurs primarily through the activation of neural signals that promote the secretion of growth hormone-releasing hormone from the hypothalamus while reducing the release of somatostatin, thereby enhancing the synthesis and release of IGF-1, which further promotes the proliferation, differentiation, and activity of osteoblasts (Lozier et al., 2018). In addition, muscle contraction activates the hypothalamic-pituitary-adrenal axis to promote testosterone secretion, which is then converted to estradiol by aromatase in adipose tissue and bone, exerting potent anti-bone-resorptive effects through estrogen receptors (Grasso et al., 2015). Also with muscle contraction, sympathetic nerve activity increases, and norepinephrine released from sympathetic nerve terminals acts directly on α2-adrenergic receptors on pancreatic β-cells, inhibiting Ca^2+^ influx and thereby directly suppressing insulin secretion, which enhances insulin sensitivity and improves the overall metabolic environment, ultimately benefiting bone metabolism (Moullé, 2022).

It has been found that proliferating cell nuclear antigen-related factor (PCLAF), as a pro-aging factor secreted by bone marrow macrophages, is highly expressed in aged mice and induces bone marrow adipocyte senescence by inhibiting AKT/mTOR signaling pathway (Xie et al., 2024). Running training can significantly reduce the levels of PCLAF and SASP factors in the bone marrow of elderly mice, reduce the expression of adipocyte senescence markers, and improve bone mass and bone metabolism indicators. Gene level verification shows that macrophage-specific knockout of PCLAF can delay age-related bone loss and adipocyte aging, further confirming that physical exercise may systematically delay bone aging through bone immune regulation pathway (Karg et al., 2017). Telomere length, as an important marker of cell senescence, is closely related to the maintenance of osteoblast function. Large sample clinical data analysis showed that accelerated telomere shortening was significantly correlated with decreased bone mineral density, and was consistent in multiple population subgroups (Valdes et al., 2007). High-intensity physical activity can improve bone loss caused by age. This finding suggests that physical exercise may maintain the proliferation and differentiation of osteocytes by slowing telomere loss, thereby protecting the integrity of bone structure. In addition, physical exercise can promote the secretion of L-β-aminoisobutyric acid (L-BAIBA) in skeletal muscle, which inhibits the expression of key transcription factors in osteoclasts by down-regulating the PI3K/AKT/NF-κB signaling pathway, and activates the NRF2 antioxidant pathway to reduce the adverse effects of reactive oxygen species accumulation on bone metabolism. As a motion response molecule, L-BAIBA not only reveals a new endocrine pathway of physical exercise regulation of bone metabolism, but also provides a potential target for the development of intervention strategies to simulate physical exercise effects (Huang et al., 2025b). Irisin is produced by physical-exercise-induced FNDC5 cleavage, which can promote osteoblast differentiation and bone formation, inhibit osteoclast formation, and improve mitochondrial function by activating the AMPK-PGC-1α pathway (Maak et al., 2021). Studies have shown that serum irisin levels are significantly increased after moderate and high intensity physical exercise, which is positively correlated with bone mineral density (Colaianni et al., 2021). Myostatin, as a negative regulation of myogenic muscle factor, its overexpression leads to muscle atrophy and bone loss. Physical exercise, especially resistance training, can significantly downregulate myostatin expression, thereby relieving the inhibition of muscle protein synthesis and bone formation (Lee, 2021).

### 4.4 Energy metabolism and mitochondrial homeostasis regulation

One of the most critical anti-aging effects of physical exercise therapy lies in its ability to reprogram cellular energy metabolism and mitochondrial function, thereby counteracting key pathogenic processes underlying osteosarcopenia. Physical exercise induces adaptive metabolic responses that restore mitochondrial homeostasis through coordinated activation of energy-sensing pathways. At the molecular level, the SIRT1–AMPK–PGC-1α signaling axis functions as a central regulator of mitochondrial biogenesis and metabolic flexibility (Chen et al., 2026). Experimental evidence from animal and cellular studies shows that both aerobic and resistance exercise activate AMPK and SIRT1, leading to upregulation of PGC-1α and enhanced mitochondrial biogenesis (Kim et al., 2018). This process improves oxidative phosphorylation efficiency and promotes fatty acid metabolism, thereby increasing ATP production and reducing metabolic stress in skeletal muscle. Human intervention studies have reported improvements in mitochondrial function and metabolic capacity following structured exercise programs, supporting the translational relevance of these findings (García-Domínguez et al., 2026).

In addition to promoting mitochondrial biogenesis, physical exercise regulates anabolic–catabolic balance through the AMPK–mTORC1 signaling network. mTORC1 is a key regulator of protein synthesis, endoplasmic reticulum function, and mitochondrial integrity. Physical-exercise-induced activation of mTORC1 promotes muscle protein synthesis and myofiber hypertrophy, while AMPK acts as a metabolic checkpoint that prevents excessive energy consumption and oxidative stress (Smiles et al., 2024). The dynamic balance between AMPK and mTORC1 signaling ensures efficient energy utilization while limiting ROS overproduction, thereby protecting cells from metabolic overload (Kim and Guan, 2019). This coordinated regulation highlights how physical exercise integrates energy sensing with structural remodeling in musculoskeletal tissues. Importantly, metabolic reprogramming induced by physical exercise extends beyond skeletal muscle and influences stem cell function and tissue regeneration. At the systems level, physical exercise-induced metabolic remodeling interacts closely with inflammatory and senescence pathways. Improved mitochondrial function reduces ROS accumulation, which in turn suppresses activation of pro-inflammatory signaling pathways such as NF-κB (Incalza et al., 2018). Reduced inflammatory signaling further limits cellular senescence and SASP secretion, thereby breaking the vicious cycle of “mitochondrial dysfunction–oxidative stress–inflammation–senescence” that drives osteosarcopenia (Zhong et al., 2023).

### 4.5 Integrated perspective: muscle contraction as a multi-pathway trigger for osteosarcopenia intervention

Taken together, the preceding sections illustrate that muscle contraction during physical exercise cannot be reduced to a single mechanical or hormonal mechanism. Instead, it functions as a coordinated trigger that simultaneously engages mechanical, paracrine, autocrine, and endocrine pathways, all of which converge to counteract osteosarcopenia. Mechanistically, mechanical loading directly activates force-sensitive signaling cascades like FAK–YAP/TAZ, Ca^2+^–calcineurin–NFAT in both muscle and bone, while the same contraction event releases a spectrum of myokines, IGF-1, and hormones that act locally via autocrine/paracrine routes and systemically via endocrine circulation. These molecular signals do not operate in isolation; they are dynamically integrated with energy-sensing networks AMPK–mTORC1–PGC-1α and immune-senescence pathways. Consequently, physical exercise restores musculoskeletal homeostasis not through a single linear mechanism but through a highly redundant, self-reinforcing network of mechanical, metabolic, endocrine, and anti-inflammatory effects. Recognizing this multi-pathway nature is essential for designing physical exercise regimens that optimally leverage the full spectrum of muscle-derived signals and for developing future interventions that mimic the integrated benefits of physical activity. This holistic understanding positions physical exercise as a paradigm of “mechanomedicine” and systemic rejuvenation, directly addressing the complexity of osteosarcopenia beyond what any single drug or isolated target can achieve. We have summarized the relevant mechanisms of physical exercise therapy in the intervention of osteosarcopenia, as shown in Table 2.

**TABLE 2:** Core mechanisms of physical exercise intervention on osteosarcopenia.

Mechanism category	Key pathways/Molecules	Core effects	References
Mechanical response	FAK/YAP/TAZ, Ca^2+^-NFAT; Wolff’s law/mechanostat	↑Bone formation ↓ bone resorption; ↑myofiber hypertrophy; ↓IL-6, TNF-α, SASP, ROS	Hammer (2015), Watson et al. (2019), Memme et al., 2021; Kwon (2024), Gehlert et al. (2015), Weng et al. (2025), de Sá Souza et al. (2022), Angulo et al. (2020), Wang et al. (2022a)
Endocrine and muscle-bone network	Irisin/FNDC5, myostatin, L-BAIBA, PCLAF, IGF-1, testosterone/estradiol	↑Irisin ↓myostatin; L-BAIBA ↓PI3K/AKT/NF-κB; ↓PCLAF&bone aging	Colaianni et al. (2015), Lozier et al. (2018), Grasso et al. (2015), Moullé, 2022; Xie et al. (2024), Karg et al. (2017), Valdes et al. (2007), Huang et al. (2025b), Maak et al. (2021), Colaianni et al. (2021), Lee (2021)
Energy metabolism and mitochondrial homeostasis	SIRT1–AMPK–PGC-1α, AMPK–mTORC1, NF-κB	↑Mitochondrial biogenesisandoxidative phosphorylation; ↓ROS&inflammation; breaks senescence cycle	Chen et al. (2026), Kim et al. (2018), García-Domínguez et al. (2026), Smiles et al. (2024), Kim and Guan (2019), Incalza et al. (2018), Zhong et al. (2023)

Calcium ion - nuclear factor of activated T-cells (Ca^2+^–NFAT). PCNA clamp associated factor (PCLAF). L-β-aminoisobutyric acid (L-BAIBA). Fibronectin type III domain-containing protein 5 (FNDC5).

## 5 Application of physical exercise in osteosarcopenia

A large amount of evidence shows that aerobic exercise and resistance exercise can effectively delay or reverse the process of cell senescence in skeletal muscle by regulating key signaling pathways, improving mitochondrial function, enhancing autophagy activity, regulating immune response and other molecular mechanisms, so as to prevent and treat sarcopenia (Fleg, 2012; Liu and Latham, 2009).

**FIGURE 3:**
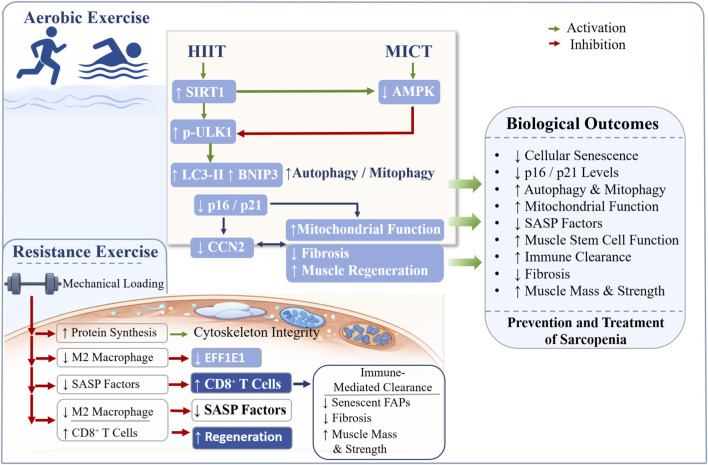
Application of physical exercise in osteosarcopenia. The abbreviations in the figure mean high-Intensity Interval Training (HIIT). Moderate-Intensity Continuous Training (MICT). Sirtuin 1 (SIRT1). Phosphorylated UNC-51-like kinase 1(p-ULK1). Microtubule-associated protein 1A/1B-light chain 3 II (LC3-II). BCL2 interacting protein 3 (BNIP3). connective tissue growth factor (CCN2). Senescence-associated secretory phenotype (SASP). Fibro-adipogenic progenitors (FAPs). Eukaryotic Elongation Factor 1ε1 (EEF1E1). Cyclin-Dependent Kinase Inhibitor 2A/1A(p16/p21). Aerobic exercise, activates the SIRT1–AMPK signaling axis, promotes ULK1 phosphorylation, and enhances autophagy/mitophagy, while suppressing p16/p21 expression. These effects contribute to improved mitochondrial function and reduced fibrosis. Resistance exercise, through mechanical loading, stimulates protein synthesis, preserves cytoskeletal integrity, and modulates the immune microenvironment by promoting M2 macrophage polarization and enhancing CD8^+^ T cell activity, thereby reducing SASP factors and facilitating muscle regeneration. Collectively, these molecular and cellular adaptations suppress cellular senescence, enhance immune-mediated clearance, and improve muscle mass and strength, ultimately contributing to the prevention and treatment of osteosarcopenia.

As a basic form of physical exercise, aerobic exercise has shown clear benefits in improving muscle aging in the elderly. Its mechanism of action is closely related to the activation of energy perception pathways. Studies have shown that aerobic exercise can reduce the expression levels of aging markers p16 and p21 in skeletal muscle of elderly mice by activating the AMPK signaling pathway (Liang et al., 2025), thereby improving muscle activity. The research has further deepened this understanding. They used the C. elegans swimming model and the elderly mouse wheel running model to find that physical exercise can activate the AdipoR1-AMPK-FOXO3a signaling axis. Activation of this pathway leads to upregulation of autophagy and mitophagy key proteins LC3-II and BNIP3, while reducing the expression of p16, ultimately improving muscle mass and reducing cell senescence (Chen et al., 2023). This not only reveals the importance of physical-exercise -regulated autophagy in combating muscle aging, but also suggests that the adipokine receptor AdipoR1 may be a potential sensor of physical exercise effects (Liu et al., 2025b). In addition, moderate-intensity continuous training (MICT) has also been shown to improve the function of muscle stem cells in the elderly. Studies have found that the expression of connective tissue growth factor (CCN2) in muscle stem cells of aged mice is significantly increased, which may be related to aging-related fibrosis. MICT intervention can significantly reduce the level of CCN2, promote muscle regeneration, and delay the process of fibrosis. The underlying mechanism may also be related to the activation of AMPK pathway (Li et al., 2024). These studies have confirmed the effectiveness of aerobic exercise in reducing cell senescence and improving muscle homeostasis by regulating core signaling pathways from the animal model level. The application of physical exercise in osteosarcopenia as shown in Figure 3.

In terms of physical exercise intensity comparison, high-intensity interval training (HIIT) shows unique advantages. A RCT of sedentary adults found that the level of eukaryotic elongation factor 1ε1 (EEF1E1) in plasma decreased significantly after HIIT, while MICT had no such effect (Dun et al., 2024). Subsequent clinical observations showed that the level of EEF1E1 in plasma of patients with sarcopenia was significantly higher than that of non-sarcopenia elderly people. It was further verified in animal experiments that HIIT was more effective than MICT in improving muscle function in aged mice, accompanied by a decrease in EEF1E1 expression. Mechanism exploration shows that HIIT may upregulate deacetylase SIRT1 and autophagy-related proteins LC3II and phosphorylated ULK, while reducing the accumulation of autophagy substrate p62, thereby more effectively removing senescent cells and related harmful factors and delaying muscle aging (Gurd et al., 2010). This series of studies combined human observation, animal experiments and molecular mechanism exploration, not only identified EEF1E1 as a potential physical exercise response and sarcopenia-related biomarker, but also emphasized the importance of physical exercise intensity in precise intervention, providing a scientific basis for the development of personalized physical exercise prescriptions.

In addition to aerobic exercise, resistance exercise is also indispensable in the prevention and treatment of sarcopenia in the elderly, and its mechanism of action focuses on different physiological pathways. Resistance exercise mainly stimulates muscle growth and strength enhancement through mechanical load. Studies have found that resistance exercise can affect signaling pathways related to energy metabolism and mitochondrial biogenesis, such as AMPK and AKT-mTOR pathways, which help to consolidate cytoskeleton tissue and reduce SASP-related inflammation levels, thereby comprehensively improving muscle mass (Zeng et al., 2020; Diniz et al., 2021). It is worth noting that dynamic resistance training is superior to isometric training in reducing aging and apoptosis markers in the muscles of elderly rats, suggesting that the specific form of resistance exercise has a significant effect on its anti-aging effect (Teixeira et al., 2020; Westcott, 2012). This shows that dynamic and progressive load may be more advantageous in designing resistance training programs for the elderly. The effect of resistance exercise on the regulation of skeletal muscle microenvironment, especially on the clearance of immune cells and senescent cells, is a research hotspot in recent years. Fibrofat progenitor cells (FAPs) play a key role in maintaining skeletal muscle homeostasis and promoting regeneration, but their own aging will be transformed into pro-fibrotic and pro-aging cells, exacerbating muscle atrophy (Giuliani et al., 2022). Studies have revealed that resistance training can effectively reduce the accumulation of senescent FAPs by inhibiting the polarization of M2 macrophages, inhibiting the release of SASP factors, and enhancing the ability of CD8 + T cells to recognize and remove senescent cells, thereby improving senile skeletal muscle atrophy (Hung et al., 2025). This study pioneered the link between the benefits of resistance exercise and immune system-mediated clearance of senescent cells, providing a new perspective for understanding how physical exercise shapes the local immune microenvironment to fight aging.

## 6 Molecular and systemic anti-aging effects of physical exercise therapy

Physical exercise is not only a simple physiological activity, but also a systematic anti-aging intervention. Its role is far beyond the local strengthening of muscles and bones, but through the multi-level molecular signal network and system steady-state remodeling to achieve the overall rejuvenation of the body function.

**FIGURE 4:**
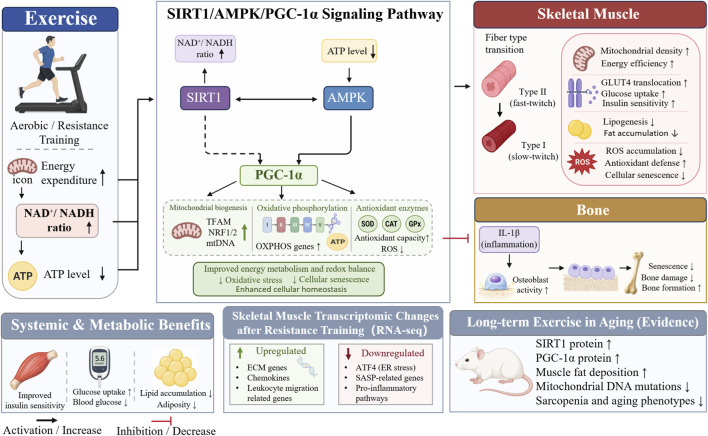
molecular and systemic anti-aging effects of physical exercise therapy. The abbreviations in the figure mean Nicotinamide adenine dinucleotide (NAD). Adenosine Triphosphate (ATP). Sirtuin 1 (SIRT1). Mitochondrial transcription factor A(TFAM). Nuclear respiratory factor 1/2 (NRF1/2). Mitochondrial DNA (mtDNA). Oxidative phosphorylation (OXPHOS). Superoxide dismutase (SOD). Catalase (CAT). Glutathione peroxidase (GPx). Reactive oxygen species (ROS). Glucose transporter type 4 (GLUT4). Interleukin 1 beta (IL-1β). Extracellular matrix (ECM). Activating transcription factor 4 (ATF4). Endoplasmic reticulum stress (ER stress). RNA sequencing (RNA-seq). Senescence-associated secretory phenotype (SASP). Schematic overview of the effects of physical exercise on skeletal muscle, bone, and systemic metabolism. Increased energy expenditure elevates the NAD^+^/NADH ratio, activating the SIRT1/AMPK/PGC-1α signaling pathway. This cascade promotes mitochondrial biogenesis, enhances oxidative phosphorylation, and upregulates antioxidant enzymes, leading to improved energy metabolism, reduced oxidative stress, and decreased cellular senescence. In skeletal muscle, physical exercise induces fiber-type transition, increases mitochondrial density and energy efficiency, enhances GLUT4 translocation and glucose uptake, reduces lipogenesis and fat accumulation, and boosts antioxidant defense. In bone, physical exercise suppresses IL-1β-mediated inflammation, increases osteoblast activity, and reduces senescence and bone damage, promoting bone formation. Systemic and metabolic benefits include improved insulin sensitivity, lower blood glucose, reduced adiposity, and decreased lipid accumulation. Transcriptomic changes after resistance training show upregulation of ECM genes, chemokines, and leukocyte migration-related genes, alongside downregulation of ATF4, SASP-related genes, and pro-inflammatory pathways. Long-term physical exercise in aging elevates SIRT1 and PGC-1α proteins, reduces muscle fat deposition and mitochondrial DNA mutations, and mitigates sarcopenia and aging phenotypes.

SIRT1 and AMPK are key molecules that regulate cell energy sensing and metabolic reprogramming (Chen et al., 2014). During physical exercise, energy expenditure and the increase of intracellular NAD/NADH ratio activate SIRT1 (Yang et al., 2022); at the same time, ATP reduction induced AMPK phosphorylation. Both of them synergistically upregulate the mitochondrial biogenesis factor PGC-1α (peroxisome proliferator-activated receptor gamma coactivator 1-alpha), promote mitochondrial synthesis, oxidative phosphorylation and antioxidant enzyme expression, thereby improving the metabolic homeostasis of skeletal muscle cells (Wang et al., 2022b). In bone tissue, the activation of SIRT1/AMPK/PGC-1α pathway can inhibit osteoblast senescence, enhance bone formation, and reduce cell damage by down-regulating IL-1β-mediated inflammatory signals (Du et al., 2021). In muscle, this pathway promotes the transformation of type II muscle fibers into fatigue-resistant type I muscle fibers, and improves mitochondrial density and energy utilization efficiency. Physical-exercise-activated AMPK also inhibits adipogenesis and promotes glucose uptake, which helps to improve insulin resistance and bone muscle energy supply. In addition, PGC-1α is also involved in regulating the expression of antioxidant systems (SOD, CAT, GPx), reducing the accumulation of reactive oxygen species ROS, and delaying cell senescence (Abu Shelbayeh et al., 2023). Studies have found that long-term moderate-intensity physical exercise significantly increased SIRT1 and PGC-1α protein levels in skeletal muscle of aged rats, and reduced muscle fat deposition and mitochondrial DNA mutation rate (Zheng et al., 2024). These results indicate that the SIRT1/AMPK/PGC-1α pathway is the molecular backbone of the anti-aging effect of physical exercise, which not only maintains the energy balance of bone and muscle, but also delays the aging process at the cellular level. The molecular and systemic anti-aging effects of physical exercise therapy as shown in Figure 4.

At the molecular level, RNA sequencing technology was used to analyze the skeletal muscle of elderly men after resistance training, revealing profound changes in its transcriptome. Studies have found that the expression of extracellular matrix (ECM) genes, chemokines and leukocyte migration-related genes related to muscle regeneration is upregulated, while the expression of activating transcription factor 4 (ATF4) and multiple SASP-related genes related to endoplasmic reticulum stress and may inhibit muscle repair is significantly decreased (Von Ruff et al., 2025). This revealed the transcriptome mechanism of resistance exercise inhibiting ATF4 signaling and aging-related pathways in elderly muscles from the whole genome scale, and clearly established the association between physical exercise intervention, muscle phenotype improvement and specific molecular pathway regulation, which laid a solid theoretical foundation for the development of precision physical exercise therapy for sarcopenia. In addition, muscle aging is often intertwined with metabolic diseases such as obesity and insulin resistance, forming a vicious circle. Studies have shown that obesity can accelerate the aging process of skeletal muscle, manifested as increased expression of aging markers (GLB1, CDKN1A, ZMAT3, CDKN2A), and decreased expression of glucose transporter GLUT4 and satellite cell marker PAX7, resulting in decreased muscle regeneration and impaired metabolic function (Podraza-Farhanieh et al., 2025). Comprehensive physical training (usually including aerobic and resistance elements) can significantly reduce cell senescence markers in skeletal muscle of obese individuals, while improving insulin sensitivity, improving blood lipid profile and reducing inflammation indicators. This effect has also been verified in cell models. By integrating human experiments and *in vitro* studies, this study strongly demonstrates that physical exercise can simultaneously improve metabolic health and muscle regeneration by reducing cell senescence in skeletal muscle, which provides strong evidence for using physical exercise intervention to simultaneously cope with aging and metabolic complications. Due to current research limitations, the molecular mechanisms underlying physical exercise therapy intervention for osteosarcopenia have not yet been fully elucidated. Further in-depth investigation of the molecular mechanisms of physical exercise therapy is of great significance for the prevention and treatment of osteosarcopenia.

## 7 Conclusion

Osteosarcopenia is a major health problem during aging, linked to increased fracture risk, muscle loss, and functional failure. Prevention is key. Physical exercise is the most economical, safe, and feasible intervention. Its core mechanism involves biomechanical and biological signaling pathways that act on bone and muscle simultaneously. Physical exercise also exerts systemic effects: it improves systemic inflammation, hormone levels, mitochondrial function, and oxidative stress, and may delay overall aging via cellular aging mechanisms.

Future research should be precise, personalized, and integrated. Priority areas include: clarifying how physical exercise factors affect SASP; revealing molecular pathways of muscle-bone crosstalk, especially effects on stem cells, mitochondrial homeostasis, epigenetics, and SASP; using genomics, proteomics, and biomarkers to tailor physical exercise prescriptions. Also, study how SASP from different sources affect bone health. Since most patients are older and have limited mobility, physical exercise interventions are often home-based, introducing uncontrollable factors for personalized guidance and remote supervision. Solutions include real-time motion guidance and data acquisition. Advanced imaging will also better assess microscopic changes in muscle and bone quality.

Addressing aging’s challenges requires exploring synergistic effects of physical exercise combined with other therapies. Combine physical activity with nutrition (protein, vitamin D, creatine), pharmacology, and physical therapies. Multimodal approaches target multiple pathways simultaneously, achieving a “1 + 1>2” synergy. Interventions should not be limited to symptomatic older people; preventive physical exercise should begin in middle age or youth to build a muscle-bone reserve, optimizing peak physiological mass and function to buffer later-life declines.

In short, physical exercise offers a new perspective for treating muscle and bone health in anti-aging. Future physical exercise programs should be integrated into public health and individualized management to effectively delay osteosarcopenia and promote healthy aging.
